# Stronger efferent suppression of cochlear neural potentials by contralateral acoustic stimulation in awake than in anesthetized chinchilla

**DOI:** 10.3389/fnsys.2015.00021

**Published:** 2015-03-02

**Authors:** Cristian Aedo, Eduardo Tapia, Elizabeth Pavez, Diego Elgueda, Paul H. Delano, Luis Robles

**Affiliations:** ^1^Departamento de Tecnología Médica, Facultad de Medicina, Universidad de ChileSantiago, Chile; ^2^Programa de Fisiología y Biofísica, ICBM, Facultad de Medicina, Universidad de ChileSantiago, RM, Chile; ^3^Departamento de Otorrinolaringología, Hospital Clínico, Universidad de ChileSantiago, Chile

**Keywords:** olivocochlear, auditory efferent, contralateral MOC reflex, CAP suppression, frequency tuning, anesthesia

## Abstract

There are two types of sensory cells in the mammalian cochlea, inner hair cells, which make synaptic contact with auditory-nerve afferent fibers, and outer hair cells that are innervated by crossed and uncrossed medial olivocochlear (MOC) efferent fibers. Contralateral acoustic stimulation activates the uncrossed efferent MOC fibers reducing cochlear neural responses, thus modifying the input to the central auditory system. The chinchilla, among all studied mammals, displays the lowest percentage of uncrossed MOC fibers raising questions about the strength and frequency distribution of the contralateral-sound effect in this species. On the other hand, MOC effects on cochlear sensitivity have been mainly studied in anesthetized animals and since the MOC-neuron activity depends on the level of anesthesia, it is important to assess the influence of anesthesia in the strength of efferent effects. Seven adult chinchillas (*Chinchilla laniger*) were chronically implanted with round-window electrodes in both cochleae. We compared the effect of contralateral sound in awake and anesthetized condition. Compound action potentials (CAP) and cochlear microphonics (CM) were measured in the ipsilateral cochlea in response to tones in absence and presence of contralateral sound. Control measurements performed after middle-ear muscles section in one animal discarded any possible middle-ear reflex activation. Contralateral sound produced CAP amplitude reductions in all chinchillas, with suppression effects greater by about 1–3 dB in awake than in anesthetized animals. In contrast, CM amplitude increases of up to 1.9 dB were found in only three awake chinchillas. In both conditions the strongest efferent effects were produced by contralateral tones at frequencies equal or close to those of ipsilateral tones. Contralateral CAP suppressions for 1–6 kHz ipsilateral tones corresponded to a span of uncrossed MOC fiber innervation reaching at least the central third of the chinchilla cochlea.

## Introduction

The cochlea of mammals has two types of sensory cells: outer (OHC) and inner (IHC) hair cells. The IHCs make synaptic contact with the afferent fibers of the auditory nerve while the OHCs are fundamentally innervated by auditory efferent axons. The auditory efferent system originates in the auditory cortex and projects mainly through two pathways. One is directed to the medial geniculate body of the thalamus, wherein it makes synapses with the afferent fibers and returns to the cortex (Winer and Prieto, 2001). The other pathway descends to the inferior colliculus and superior olivary complex finally projecting through medial olivocochlear (MOC) fibers to both cochleae (Vetter et al., 1993). The MOC fibers are myelinated axons that originate in the ventral nucleus of the trapezoid body and that segregate into crossed and uncrossed fibers that make synaptic contact with OHCs in the contra- and ipsilateral cochleae, respectively. The lateral olivocochlear (LOC) system is constituted by unmyelinated neurons that project mainly to the ipsilateral cochlea making synaptic contacts beneath IHCs (Warr and Guinan, 1979). Based on the available experimental evidence several possible roles have been assigned to the auditory efferent system, such as: reduction of the masking effect produced by noise or tones (Kawase et al., 1993), a protective role in preventing loss of sensitivity caused by exposure to high-intensity sounds (Rajan, 1995), modulation of responses to auditory stimuli within the sleep-wake cycle (Velluti, 1997), modulation of cochlear sensitivity according to attentional processes (Delano et al., 2007) and a balancing effect on interaural sensitivity (Darrow et al., 2006). (For review see Guinan, 2006; Robles and Delano, 2008).

It has been shown that stimulation of the MOC efferent system by means of electrical pulses delivered at the floor of the fourth ventricle produces a decrease in the amplitude of auditory-nerve compound action potentials (CAP) and a concomitant increase in cochlear microphonic (CM) potentials (Galambos, 1956; Fex, 1959; Desmedt and Monaco, 1961; Gifford and Guinan, 1987; Elgueda et al., 2011). Auditory efferent fibers can also be activated by acoustic stimulation of the contralateral ear producing a decrease in single auditory-nerve fiber and CAP responses to ipsilateral tones (Buño, 1978; Liberman, 1989; Warren and Liberman, 1989a). This efferent suppression produced by contralateral sounds, known as the “contra MOC reflex” (de Venecia et al., 2005) is mediated by MOC uncrossed fibers. In all species studied so far uncrossed MOC fibers comprise a smaller percentage of the total than the crossed MOC fibers (Robles and Delano, 2008). However, there are significant differences in the distribution of MOC fibers among species. The finding that the chinchilla displays the lowest percentage of uncrossed fibers among all studied mammals has raised questions about the strength and frequency distribution of the contra MOC reflex in this species (Iurato et al., 1978; Azeredo et al., 1999). In fact, as reported in Azeredo et al. (1999), the relative paucity of MOC uncrossed fibers and their strong apical bias may have been contributing factors in the early unsuccessful attempts to demonstrate CAP suppression with contralateral noise in the chinchilla.

The aims of this work are: to determine the strength of the contra MOC reflex at different locations along the cochlea in the chinchilla, to ascertain whether the suppression due to contralateral tones is tuned to the ipsilateral-tone frequency and to compare the CAP and CM efferent effects in the awake and anesthetized chinchilla.

## Materials and methods

### Animals

Seven adult Chinchillas (*Chinchilla laniger*) weighing between 400 and 700 grams were used. All procedures involving animals were made in accordance with NIH Guidelines for the Care and Use of Laboratory Animals, publication No. 86–23, revised 1996, and were approved by the Institutional Bioethics Committee (Comité de Bioética de Investigación en Animales, Facultad de Medicina, Universidad de Chile, permit number CBA #0262). All surgeries were performed under ketamine and xylazine anesthesia, and every effort was made to minimize animal suffering.

### Surgery

Chinchillas were premedicated with atropine (0.04 mg/kg I.M.), xylazine (3–8 mg/kg I.M.) and then anesthetized with ketamine (20–40 mg/kg, I.M.). After the surgery, they were treated with analgesics (Ketofen®, 3 mg/kg I.M.) and antibiotics (Baytril®, 5 mg/kg I.M.) every 12 h for 5 days. A craniotomy was performed in the lateral tympanic bulla and a cochlear electrode (Nichrome® 200 μm diameter) was placed on the round window niche membrane and connected to an external connector that was chronically implanted on the animal’s skull. The middle-ear ossicles remained intact to preserve the physiologic conduction of sound to the cochlea. During surgery rectal temperature was maintained constant at 35–37°C by means of a heating pad. All surgical procedures were performed under a microscope (Zeiss®, OpMi-1) with magnification of up to 40x.

### Stimulation protocol and data acquisition

The effects produced by contralateral stimulation on cochlear potentials were measured in all chinchillas, first in awake and later in anesthetized condition. For the measurements in the awake condition the animals were kept in a custom-made motion restrainer that limited their movements with minimum discomfort in sessions of less than 45 min duration. The restrainer device consisted in a cloth hammock in which the animal was suspended and a padded ring that was adjusted around its neck to restrict the head movements. A week before the surgical electrode implantation, the animals were trained during 3–5 days in sessions not exceeding 45 min to stay quiet on the restrainer device.

**Figure 1:**
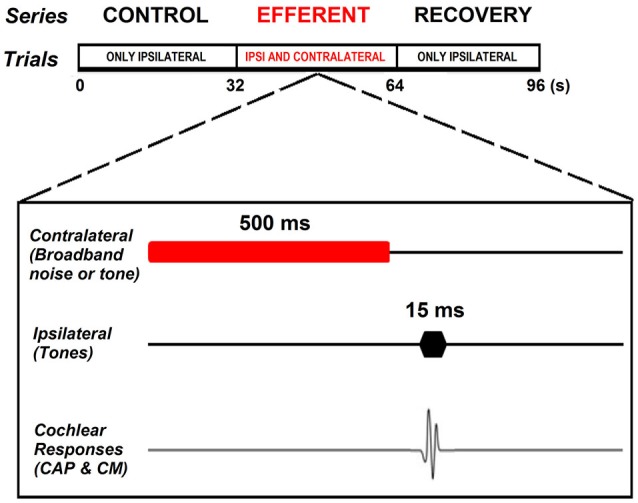
**Experimental paradigm**. Each stimulation sequence consisted of three consecutive series of 32 stimuli presented at 1 Hz rate: control, efferent and recovery. Control series consisting of ipsilateral tones (15 ms duration) presented alone. Efferent series in which the ipsilateral tone (15 ms duration) was preceded by a contralateral tone or noise (500 ms duration) followed by a silent period (15 ms duration). Recovery series in which the ipsilateral tones (15 ms duration) were again presented alone.

All experiments were performed in a double-walled sound-attenuating room, isolated from external noises and vibrations. The same stimulation protocol was used in anesthetized and awake animals. Acoustic stimuli were digitally generated with a National Instruments® (PCI-6071E) multifunction data acquisition device, attenuated by programmable attenuators (PA-5, Tucker Davis Technologies®, TDT System 3) and delivered through insertion phones (EC1 electrostatic speakers, TDT). Cochlear potentials were recorded in response to tones at different sound pressure levels (40–80 dB SPL for ipsilateral stimuli and 50–75 dB for contralateral stimuli) and frequencies ranging from 1–6 kHz. Acoustical stimuli were presented with alternated polarity in order to allow us to separate CAPs from CMs. Responses to stimuli of each polarity were independently averaged and later added together to cancel CMs and isolate CAP responses. Each stimulus sequence consisted of 3 consecutive series of 32 stimuli presented at a 1 Hz rate: control, efferent and recovery. In the control series the ipsilateral tones (15 ms duration) were presented alone. In the efferent series the ipsilateral tones (15 ms duration) were preceded by a contralateral tone or noise (500 ms duration) followed by a silent period (15 ms duration). In the recovery series the ipsilateral tones (15 ms duration) were again presented alone (Figure 1). The noise stimulus was digitally generated and filtered (0.5–32 kHz) uniform white noise (LabWindows®, white noise subroutine). Cochlear potentials were amplified (10,000X), filtered (0.1–10 kHz) and digitized (40,000 points/s) by a National Instruments® (PCI-6024E) card. CAP amplitudes were measured between the maximum and minimum of N1 and P1 waves (first and second peaks) of the average of 32 responses. Subtraction of the averaged responses to stimuli of the two polarities allowed the computation of the CM amplitudes by performing a Fast Fourier Transform (FFT) in a 12.8 ms window that excluded the CAP response. Efferent effects on CAP and CM responses were expressed in dB referred to the control amplitudes. In the case of efferent effects on CMs that were fairly small we used a threshold of 0.5 dB for CM amplitude changes. This threshold was established by measuring, in two animals, the variability of repeated measures of CM amplitude for the same parameters of ipsilateral stimulation, without efferent activation. The variability obtained ranged from 1 to 3 % or 0.1 to 0.25 dB, therefore to make sure the changes were not due to the variability of the responses we set a threshold equal twice the maximum variability. In two chinchillas at the end of the experiment we injected tetrodotoxin (TTX, 3 μM), a powerful neurotoxin that blocks voltage-dependent Na^+^ ion channels, through the round window of the contralateral cochlea to block neural responses and repeated some of the measurements of contralateral-ear suppression. Data analysis was performed using custom-made C programs (LabWindows®). The significance of the differences between the experimental and control series was checked using *t*-tests or ANOVA for normal distributed data or the Mann-Whitney test (SigmaPlot® v12.5) for non-normal data (as evaluated by the Shapiro-Wilk test).

## Results

**Figure 2:**
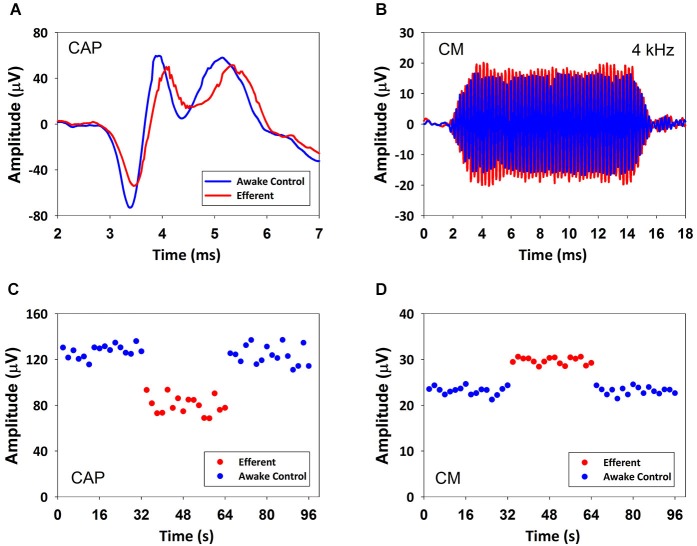
**CAP suppression and CM increase produced by contralateral acoustical stimulation in an awake chinchilla**. **(A)** Average CAP traces of 32 responses to ipsilateral tones in absence (blue) and presence (red) of contralateral acoustical stimulation. **(B)** Average CM traces of 32 responses to ipsilateral tones in absence (blue) and presence (red) of contralateral acoustical stimulation. **(C)** CAP response amplitudes in repeated trials in absence (blue) and presence (red) of contralateral acoustical stimulation. Each symbol represents the average CAP amplitude (N1 to P1 in μV) of two consecutive trials. Significant CAP reductions were obtained with contralateral acoustical stimulation (two tailed *t*-test, *T*_(30)_ = 19.618, *p* = 1.157*10^−18^). **(D)** CM response amplitudes in repeated trials in absence (blue) and presence (red) of contralateral acoustical stimulation. Each symbol represents the average CM amplitude of two consecutive trials. Amplitudes (rms in μV) were obtained by fast Fourier transform (FFT). Significant CM enhancements were obtained with contralateral acoustical stimulation (two tailed *t*-test, *T*_(30)_ = −22.329, *p* = 3.031*10^−20^). In all panels ipsilateral tones were 4 kHz at 60 dB SPL and contralateral broad-band noise was at 55 dB SPL.

The effect of contralateral broad-band noise on the magnitudes of the CAP and CMs generated in response to ipsilateral tones (1–6 kHz) was measured in seven chinchillas in awake and anesthetized condition. CAP-amplitude reductions produced by contralateral noise ranged from 1 to 7 dB in anesthetized and from 1 to 10 dB in awake animals. In contrast, no measurable contralateral-sound effects were obtained on CMs in anesthetized chinchillas. In awake state efferent effects on CM were found in only three chinchillas that displayed CM amplitude increases ranging from 0.5 to 1.9 dB. Figure 2 depicts the effects of contralateral broad-band noise on CAP and CM responses obtained in the awake chinchilla that displayed the largest CM increases, concomitant to CAP reductions. Since contralateral-sound effects on CM potentials were obtained in only three awake animals the following description and analysis of results will be limited to the effects on CAP responses.

**Figure 3:**
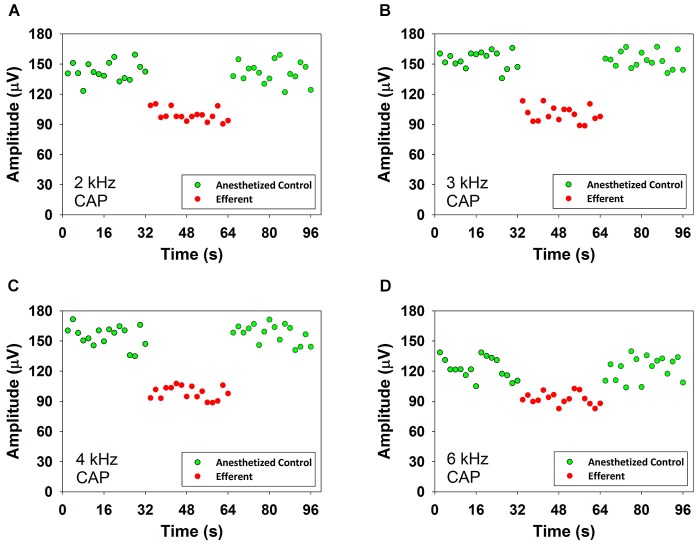
**CAP suppression produced by contralateral acoustical stimulation in an anesthetized chinchilla**. Reductions of CAP amplitudes in response to ipsilateral tones obtained in the presence of contralateral broad-band noise (55 dB SPL). Each symbol represents the average CAP amplitude of two consecutive trials. The red circles correspond to responses to ipsilateral tones preceded by contralateral efferent stimulation. The green circles correspond to responses to ipsilateral tones alone, before and after contralateral efferent stimulation. Panel **(A)**: 2 kHz at 47 dB SPL, panel **(B)**: 3 kHz at 50 dB SPL, panel **(C)**: 4 kHz at 44 dB SPL and panel **(D)**: 6 kHz at 40 dB SPL. Significant CAP reductions were obtained with contralateral acoustical stimulation at 2 kHz (two tailed *t*-test, *T*_(30)_ = 15.324, *p* = 9.919*10^−16^), 3 kHz (two tailed *t*-test, *T*_(30)_ = 18.924, *p* = 3.150*10^−18^), 4 kHz (two tailed *t*-test, *T*_(30)_ = 9.920, *p* = 5.509*10^−11^) and 6 kHz (two tailed *t*-test, *T*_(30)_ = 18.391, *p* = 6.957*10^−18^).

Figure 3 shows reductions produced by contralateral noise (40–50 dB SPL) in the amplitude of CAP responses to ipsilateral tones at four frequencies in one anesthetized chinchilla. In the figure green and red symbols indicate CAP amplitudes in absence and presence of contralateral stimulation, respectively. As exemplified in this figure, in all cases the presence of the contralateral sound produced an abrupt decrease in CAP amplitude that could be seen in the first CAP response after the onset of the contralateral stimulation series and an equally abrupt return to the control value at the offset of stimulation.

**Figure 4:**
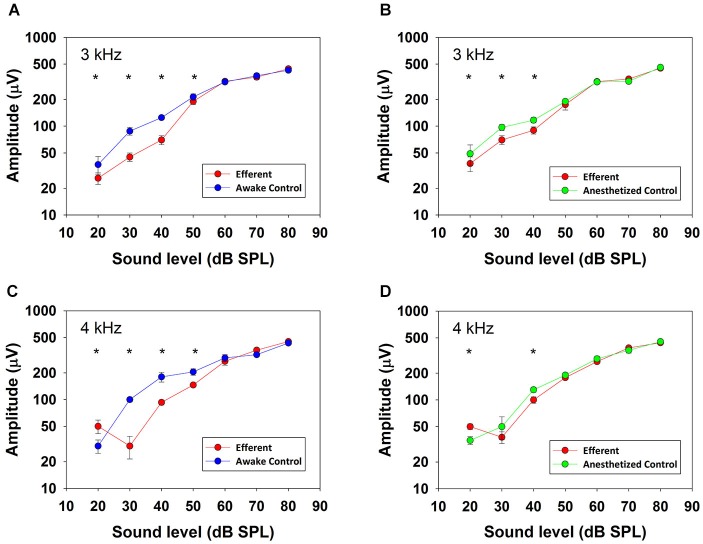
**CAP input–output curves**. CAP amplitudes obtained in one chinchilla without (blue and green) and with (red) contralateral acoustical stimulation (broad-band noise at 50 dB SPL) in awake (blue) and anesthetized (green) condition. Vertical lines indicate standard deviations. Efferent-activation produced by contralateral stimulation is more effective at low ipsilateral stimulus intensities and efferent reductions are higher in awake than in anesthetized animals. Asterisks indicate statistically significant differences. Panel **(A)**: 3 kHz, 20 dB: (Mann-Whitney, *U*_(32)_ = 30.0, *T* = 1522, *p* < 0.001); 3 kHz, 30 dB: (Mann-Whitney, *U*_(32)_ = 0.0, *T* = 528.0, *p* < 0.001); 3 kHz, 40 dB: (Mann-Whitney, *U*_(32)_ = 0.0, *T* = 1522.0, *p* < 0.001); 3 kHz, 50 dB: (Mann-Whitney, *U*_(32)_ = 126.0, *T* = 1426.0, *p* < 0.001). Panel **(B)**: 3 kHz, 20 dB: (Mann-Whitney, *U*_(32)_ = 170.0, *T* = 1382.0, *p* < 0.001); 3 kHz, 30 dB: (Mann-Whitney, *U*_(32)_ = 5.0, *T* = 1547.0, *p* < 0.001); 3 kHz, 40 dB: (Mann-Whitney, *U*_(32)_ = 0.0, *T* = 528.0, *p* < 0.001). Panel **(C)**: 4 kHz, 20 dB: (Mann-Whitney, *U*_(32)_ = 266.0, *T* = 1286.0, *p* < 0.001); 4 kHz, 30 dB: (Mann-Whitney, *U*_(32)_ = 0.0, *T* = 528.0, *p* < 0.001); 4 kHz, 40 dB: (Mann-Whitney, *U*_(32)_ = 0.0, *T* = 528.0, *p* < 0.001); 4 kHz, 50 dB: (Mann-Whitney, *U*_(32)_ = 179.0, *T* = 707.0, *p* < 0.001). Panel **(D)**: 4 kHz, 20 dB: (Mann-Whitney, *U*_(32)_ = 266.0, *T* = 1286.0, *p* < 0.001); 4 kHz, 40 dB: (Mann-Whitney, *U*_(32)_ = 2.0, *T* = 1550.0, *p* < 0.001).

Figure 4 shows input-output curves of CAP amplitudes in response to ipsilateral tones at frequencies of 3 and 4 kHz and intensities from 20 to 80 dB SPL obtained in absence and presence of contralateral broad-band noise (50 dB SPL; red curves) in one awake (blue) and anesthetized (green) chinchilla. All of the curves show a monotonic CAP amplitude increase with increasing stimulation intensity that in some cases reaches saturation at levels above 80 dB SPL (not shown). The figure compares CAP suppressions obtained at the two ipsilateral tone frequencies that displayed the strongest efferent effects. At both frequencies, in awake condition, all amplitude differences reached statistical significance for ipsilateral tones <60 dB SPL, while in anesthetized condition, most amplitude differences were significant for ipsilateral tones <50 dB SPL.

**Figure 5:**
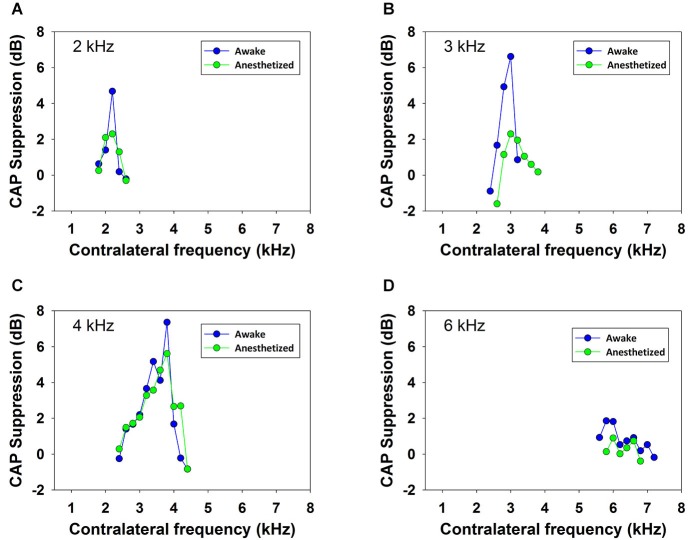
**Frequency tuning of ipsilateral CAP reduction produced by contralateral tone stimulation**. Efferent reduction of CAP amplitudes produced by the presence of contralateral acoustical stimulation in four animals, in awake (blue) and anesthetized (green) condition, for ipsilateral tones at frequencies of 2, 3, 4 and 6 kHz. Intensities of the ipsilateral and contralateral tones were, panel **(A)**: 55 and 69 dB SPL, panel **(B)**: 58 and 64 dB SPL, panel **(C)**: 56 and 62 dB SPL and panel **(D)**: 53 and 58 dB SPL.

**Figure 6:**
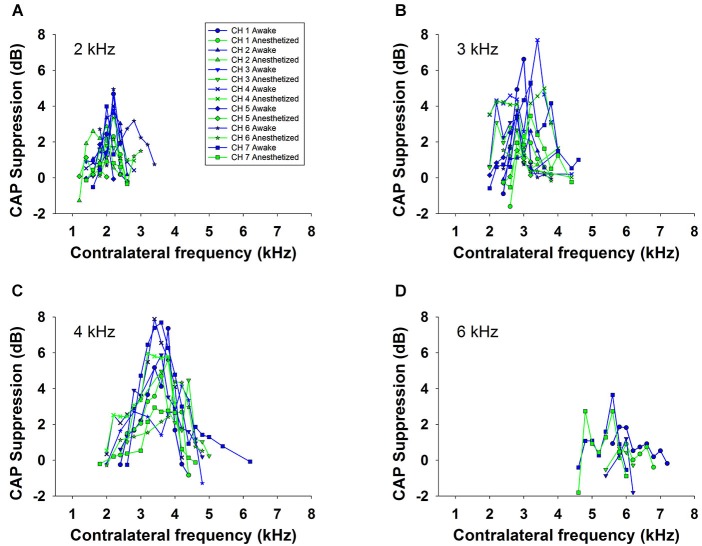
**Frequency tuning of ipsilateral CAP reduction produced by contralateral tone stimulation in all animals**. Superposition of the curves of CAP suppression produced by contralateral acoustical stimulation in all animals, in awake (blue) and anesthetized (green) condition, for ipsilateral tones at, panel **(A)**: 2 kHz, panel **(B)**: 3 kHz, panel **(C)**: 4 kHz and panel **(D)**: 6 kHz. Intensities of the tones were: 50 to 60 dB SPL for the ipsilateral and 60 to 70 dB SPL for the contralateral tones.

**Figure 7:**
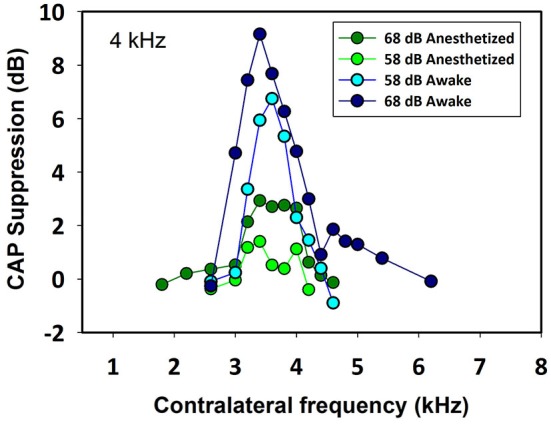
**Frequency tuning curves of ipsilateral CAP reduction produced by contralateral tones at two intensities in an awake and anesthetized chinchilla**. The magnitude and extent of the CAP reduction depend on the contralateral stimulus frequency and intensity. In this case, for a 4 kHz ipsilateral tone (48 dB SPL) the greatest CAP reductions were obtained for contralateral frequencies between 3400 and 4000 Hz. As in all other animals, the efferent effect was better tuned and stronger in awake than in anesthetized condition. Awake vs. anesthetized, 68 dB SPL (Mann-Whitney, *U*_(32)_ = 0.0, *T* = 392, *p* < 0.001), 58 dB SPL (Mann-Whitney, *U*_(32)_ = 0.0, *T* = 392, *p* < 0.001); 68 dB SPL vs. 58 dB SPL, awake (Mann-Whitney, *U*_(32)_ = 45.0, *T* = 181, *p* = 0.002), anesthetized (Mann-Whitney, *U*_(32)_ = 0.0, *T* = 136, *p* < 0.001).

Contralateral stimulation with pure tones also produced CAP reductions in anesthetized as well as in awake animals; however, the magnitude of the effect was highly dependent on the frequency of the contralateral tone. Figure 5 displays examples of the reductions of CAP responses to ipsilateral tones at four frequencies (2–6 kHz) produced by contralateral tones of different frequencies in awake and anesthetized animals. The efferent suppression produced by contralateral tones was always tuned to a frequency equal or close to the ipsilateral stimulus tone and, as in the case with contralateral noise, it was stronger in awake than in anesthetized animals. To give an idea of the differences in frequency tuning of efferent suppression observed in the different animals we present in Figure 6 the superposition of the curves of CAP reduction obtained, at four ipsilateral frequencies, for all contralateral frequencies tested in all animals in awake and anesthetized condition (intensity levels: 50–60 dB SPL ipsilateral and 60–70 dB SPL contralateral tones). Significant effects were obtained with frequency and with awake or anesthesia conditions as assessed by a two-way ANOVA (ipsilateral frequency vs. awake/anesthesia as factors): awake vs. anesthesia: *F*_(1)_ = 5.681, *p* = 0.018; ipsilateral frequency: *F*_(3)_ = 14.199, *p* < 0.001. However, there was no interaction between these factors: *F*_(3)_ = 0.182, *p* = 0.908. The magnitudes and extent of the CAP reductions were also dependent on the intensity of the contralateral tone. Figure 7 compares the magnitudes of CAP suppressions obtained in one animal, for a 4 kHz ipsilateral tone in anesthetized and awake condition, with contralateral tones at frequencies ranging from 1800 to 6200 Hz and intensities at 58 and 68 dB SPL. In both conditions, awake and anesthetized, the efferent suppression effects were stronger and extended on a wider range of frequencies for the more intense contralateral tones.

**Figure 8:**
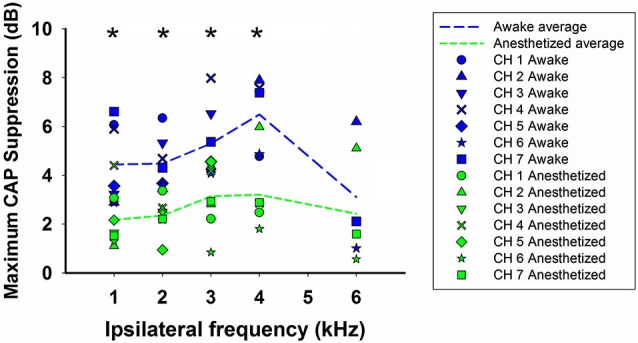
**Summary of efferent CAP suppression in awake and anesthetized animals**. Symbols depict maximum CAP supressions produced by contralateral stimulation, in each animal, at each ipsilateral frequency in awake (blue) and anesthetized (green) condition. The dashed lines display the average of the maximum CAP reductions produced by contralateral tones for all animals at each ipsilateral frequency in awake (blue) and anesthetized condition (green). Intensities of ipsilateral and contralateral tones were 50–60 and 60–70 dB SPL, respectively. Asterisks indicate statistical significance.

We have compared the CAP suppressions obtained in awake and anesthetized animals for 1–6 kHz ipsilateral frequencies and similar stimulation intensity levels: 50–60 dB SPL ipsilateral tones and 60–70 dB SPL contralateral tones. Figure 8 depicts the maximum CAP reductions produced by contralateral stimulation, in each animal, at each ipsilateral frequency in awake and anesthetized condition (blue and green symbols, respectively). The figure also displays the average of the maximum CAP reductions produced by contralateral tones for all animals at each ipsilateral frequency in awake and anesthetized condition (blue and green dashed lines, respectively). As shown in the figure, the average CAP suppressions produced by contralateral tones in all chinchillas were significantly larger, at almost all frequencies, in the awake than in the anesthetized condition (Mann-Whitney test, 1 kHz: *U*_(7)_ = 6.0, *T* = 71.0, *p* = 0.017; 2 kHz: *U*_(7)_ = 0.0, *T* = 77, *p* < 0.001; 3 kHz: *U*_(7)_ = 7.5, *T* = 69.5, *p* = 0.026; 4 kHz: *U*_(5)_ = 2.0, *T* = 38, *p* = 0.032; 6 kHz: *U*_(3)_ = 3.0, *T* = 12, *p* = 0.7).

**Figure 9:**
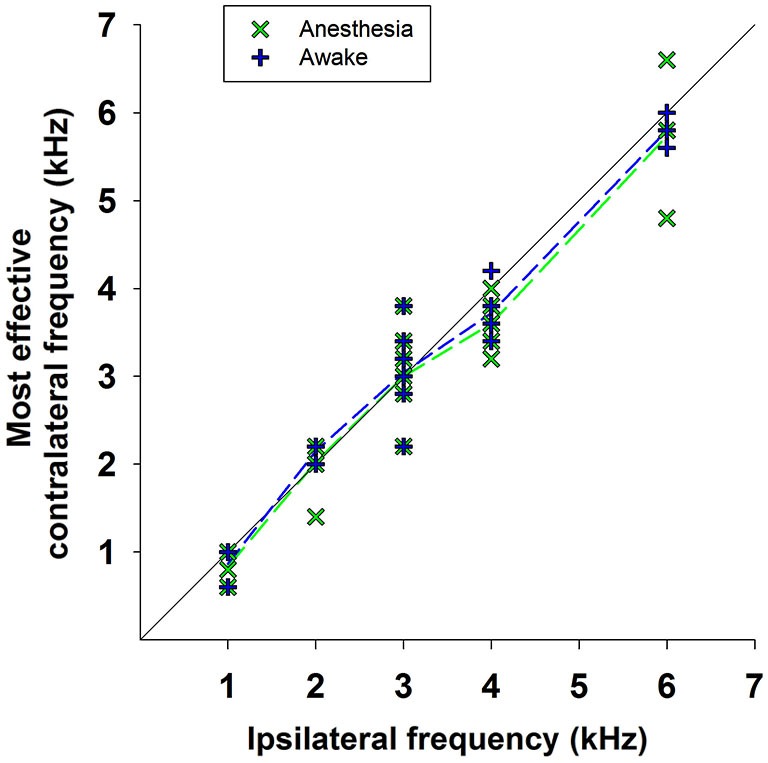
**Most effective contralateral suppressor frequencies vs. ipsilateral frequencies**. Relationship between the frequencies of the most effective contralateral suppressors and the ipsilateral tones for all data obtained in awake (blue symbols) and anesthetized (green symbols) animals. The dashed lines indicate the average values of the most effective contralateral suppressors frequencies for all measurements at each ipsilateral frequency in awake (blue dashed line) and anesthetized (green dashed line) animals.

**Figure 10:**
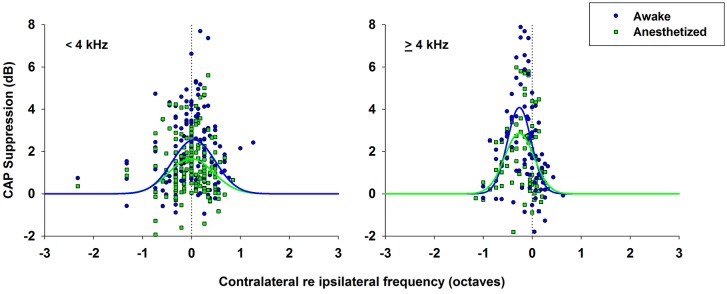
**CAP suppression as a function of contralateral suppressor frequency re ipsilateral frequency**. CAP suppressions as a function of the difference between contralateral frequencies and ipsilateral frequencies (in octaves) for all data in awake (blue circles) and anesthetized (green squares) animals. Normal distributions fitted to data, awake (blue line), anesthetized (green line). (Left) CAP suppression for ipsilateral frequencies <4 kHz. Awake, $f\left(x\right)=2.56\;e^{-0.5}{\left(\frac{x-0.0483}{0.435}\right)}^{2}$; anesthetized, $f(x)=1.66\;e^{-0.5}{\left(\frac{x+0.0067}{0.435}\right)}^{2}$. (Right) CAP suppression for ipsilateral frequencies ≥ 4 kHz. Awake, $f(x)=4.084\;e^{-0.5}{\left(\frac{x+0.262}{0.245}\right)}^{2}$; anesthetized, $f(x)=2.868\;e^{-0.5}{\left(\frac{x+0.263}{0.301}\right)}^{2}$.

As mentioned above, the efferent CAP suppression produced by contralateral tones was always tuned in frequency; that is, there was a most effective contralateral frequency, close or equal to the ipsilateral-tone frequency, that produced maximal suppression (Figures 5–7). Figure 9 shows the relationship between the most effective contralateral frequencies and ipsilateral frequencies for all data obtained in awake and anesthetized animals (blue and green symbols, respectively). In both conditions, the most effective contralateral suppressor frequencies for ipsilateral frequencies <4 kHz were nearly equal to the ipsilateral frecuencies, while for ipsilateral frequencies ≥4 kHz they were consistently lower than ipsilateral frequencies, as shown by the average values of the most effective contralateral frequencies (dashed lines). This different relation between most effective contralateral and ipsilateral frequencies is also illustrated in Figure 10, which depicts CAP suppressions as a function of the difference (in octaves) between most effective suppressor and ipsilateral frequencies for the data obtained in all animals, but segregated into two groups according to the values of ipsilateral frequency. In awake, as well as, in anesthetized animals CAP suppressions display distributions that, for ipsilateral frequencies <4 kHz are centered at most effective suppressor frequencies equal to ipsilateral frequencies, while for ipsilateral frequencies ≥4 kHz exhibit an offset of −0.26 octaves between most effective suppressor and ipsilateral frequencies. The figure shows that CAP-suppression curves for ipsilateral frequencies ≥4 kHz display higher tuning than those of ipsilateral frequencies <4 kHz, but that there are no changes in the pattern of tuning with and without anesthesia.

**Figure 11:**
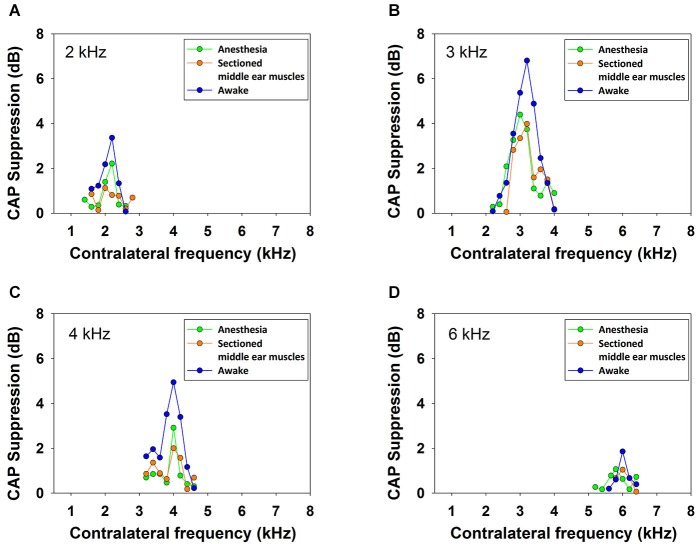
**Frequency tuning curves in awake and anesthetized chinchilla with intact and sectioned middle-ear muscles**. Efferent reduction of CAP amplitudes produced by contralateral acoustical stimulation (70 dB SPL) in one animal, awake (blue), anesthetized (green) and anesthetized with detached middle-ear muscles (orange), for ipsilateral tones (50 dB SPL) at, panel **(A)**: 2 kHz, panel **(B)**: 3 kHz, panel **(C)**: 4 kHz and panel **(D)**: 6 kHz. There were no significant differences between the results obtained in the anesthetized animal before and after middle-ear muscles detachment.

In order to discard any possibility that the reduction in CAP amplitude could be produced by activation of the middle-ear reflex, in one anesthetized animal the experimental protocol for frequency tuning of CAP suppression was repeated after section of the tensor tympani muscle and detachment of the stapedius muscle from its insertion. As shown in Figure 11, at four ipsilateral frequencies, no significant differences were found in contralateral CAP suppression for the magnitudes obtained before and after the middle-ear muscle section.

**Figure 12:**
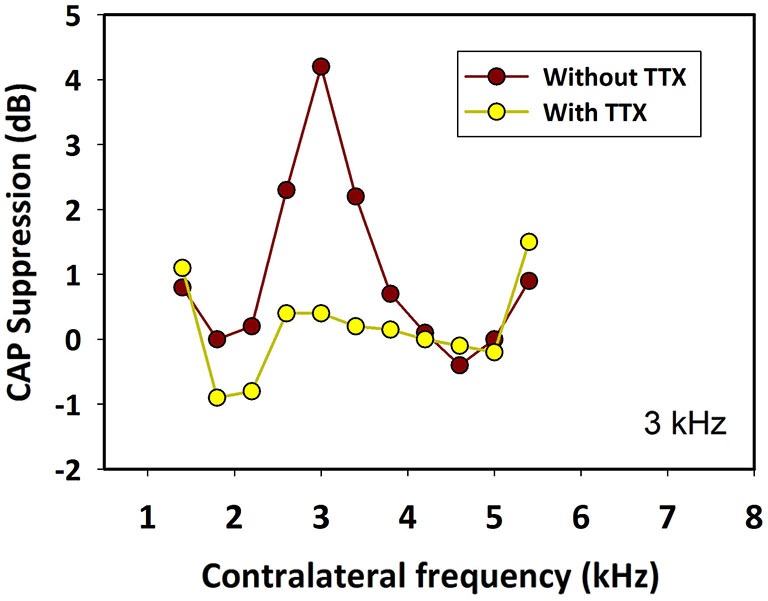
**Effect of tetrodotoxin (TTX) in the contralateral cochlea**. Ipsilateral CAP-reduction tuning curves before (brown) and after (yellow) injection of tetrodotoxin into the contralateral cochlea. The toxin abolished contralateral neural responses and their suppressive effect on ipsilateral CAPs. Ipsilateral tones at 3 kHz and 50 dB SPL. Contralateral tones at 70 dB SPL.

In addition, we performed two kinds of controls to show that CAP suppression was mediated by MOC neurons activated from the contralateral ear and was not due to ipsilateral forward masking or ipsilateral activation of MOC fibers produced by crosstalk of contralateral stimuli to the ipsilateral ear. First, in two animals we injected tetrodotoxin (TTX), a powerful neurotoxin blocker of voltage-dependent Na^+^ ion channels, into the contralateral cochlea. After minutes of the TTX injection, in both animals, the toxin abolished the contralateral-ear neural responses and also their suppressive effect on ipsilateral CAPs (Figure 12). The disappearance of the CAP suppression after the TTX contralateral injection showed that the effect was mediated by the neural response of the contralateral ear. Second, in one chinchilla we determined that the interaural attenuation was higher than 40 dB at all frequencies of contralateral stimulation. Then, after measuring the CAP suppression produced by a contralateral tone, we evaluated the CAP suppression produced by an ipsilateral tone at an intensity 40 dB lower than that of the contralateral tone. CAP suppression was found for the contralateral tone stimulation, but not for the 40 dB-lower ipsilateral tone, thus showing that the CAP suppression measured in the experiments was exclusively produced by MOC neurons activated from the contralateral ear.

## Discussion

The effect of contralateral sound stimulation on cochlear responses has been studied recording auditory-nerve fiber and CAP responses and distortion product otoacoustic emissions (DPOAEs) in cat, guinea pig and mice (Buño, 1978; Liberman, 1989; Warren and Liberman, 1989a,b; Puel and Rebillard, 1990; Popelár et al., 2001; Boyev et al., 2002; Chambers et al., 2012). In the cat it has been shown that contralateral tones and broad-band noise produce a decrease in the responses of single auditory-nerve fibers to ipsilateral tones that is greatest for auditory fibers with CF near 2 kHz and that could reach up to about 70% (Warren and Liberman, 1989a). Severing of the olivocochlear bundle at the internal auditory meatus completely eliminated the suppressive effects of contralateral sound, while severing only the crossed olivocochlear fibers did not eliminate them, thus indicating that these contralateral-sound suppressive effects were mediated by uncrossed olivocochlear fibers (Warren and Liberman, 1989a). Suppressive effects of contralateral noise and tones on CAP responses have also been reported in anesthetized cats and guinea pigs (Liberman, 1989; Puria et al., 1996; Larsen and Liberman, 2009). These suppressive effects, as in the case of auditory-nerve responses, were mediated by uncrossed olivocochlear fibers. Furthermore, Liberman (1989) recording CAP and single olivocochlear-fiber responses in the same animals obtained good correlation between the strength of CAP suppression and the sound-evoked discharge rates of single olivocochlear neurons. All of this evidence indicates that the effects of contralateral sound stimulation are dependent on the uncrossed MOC efferent fibers.

As mentioned in the Introduction, among all species studied, the chinchilla is the one that presents the lowest percentage of uncrossed MOC fibers (about 20%; Azeredo et al., 1999). Since these are the efferent fibers comprising the neural pathway that mediates contralateral sound suppression, their scarcity in the chinchilla raised doubts about the strength and frequency distribution of the contralateral-sound suppression that could be found in this species. In fact, early works in chinchilla reported unsuccessful attempts to suppress CAP responses and DPOAEs with contralateral noise stimulation (Azeredo et al., 1999). In contrast with the outcome of those early attempts, more recent studies in chinchilla have succeed in finding contralateral-sound suppressive effects on ipsilateral DPOAEs (James et al., 2005; Harrison et al., 2008; Wolter et al., 2014) and in the present work we are reporting consistent contralateral-sound suppression of CAP responses in the seven chinchillas studied.

### Efferent modulation of cochlear responses in chinchilla

Two types of efferent effects on cochlear responses with different temporal courses have been described: fast effects that have time constants of tens of milliseconds and slow effects that have time constants three orders of magnitude larger (Sridhar et al., 1995; Cooper and Guinan, 2003). Although our experimental paradigm allowed the assessment of both fast and slow MOC effects, we only found fast amplitude changes in cochlear potentials. In all animals CAP amplitudes abruptly decreased at the onset of the contralateral stimulation series and abruptly returned to control values at the offset, without evidence of slower changes of amplitude during the 32 s stimulation period. Similar abrupt amplitude increases, instead of decreases, were observed in the CM responses of the three awake chinchillas that exhibited CM efferent effects (see Figures 2, 3). However, it is possible that our contralateral stimulation period could have been too short to produce slow effects, as other studies using other contralateral stimulation paradigms in chinchilla have reported slow efferent effects after 1 min of contralateral stimulation (Bowen et al., 2014).

We have found in chinchilla large CAP-amplitude suppressions produced by contralateral broad-band noise as well as by contralateral tones. But, a comparison of the magnitudes of contralateral CAP suppression obtained in our study in chinchilla with values reported in other species is difficult because of the dependance of suppression on the different paradigms and parameters of stimulation used by different authors. However, in spite of the lower percentage of uncrossed MOC fibers in chinchilla, our highest values of suppression of about 10 dB (computed as equivalent or effective attenuation; Desmedt, 1962; Liberman, 1989) are similar to those reported in cat (Liberman, 1989).

We were able to demonstrate contralateral CAP suppression for ipsilateral tones with frequencies in the range of 1–6 kHz. This would indicate a span of efferent innervation reaching at least the central third of the cochlea, according to the chinchilla cochlea frequency-position map (Müller et al., 2010). The only anatomical study on efferent fiber distribution available in chinchilla shows that uncrossed MOC fibers have a strong bias toward the more apical cochlear regions: no uncrossed MOC fibers were found in the most basal cochlear region, only 12% of MOC fibers in the basal turn, about 30% in the second turn and 57% in the apical turn (Iurato et al., 1978). This anatomical distribution of uncrossed MOC fibers is compatible with our finding of contralateral suppression for ipsilateral tones with frequencies of 1–6 kHz. The largest suppressions produced by contralateral tones in this study were for ipsilateral frequencies at 3 and 4 kHz. In the cat the largest CAP suppressions were found for an ipsilateral frequency of 1.5 kHz (Liberman, 1989). In both cases maximal suppressions occurred for frequencies corresponding to locations in the mid turn of the cochlea (Greenwood, 1990; Müller et al., 2010).

As mentioned in Results, we consistently found that contralateral sounds produced substantial CAP-amplitude reductions and no measurable effects on CMs in anesthetized chinchillas and small CM enhancements in only three of the seven awake chinchillas. This absence or weakness of efferent effects on CMs in the present results is consistent with results obtained in a previous study in which we electrically stimulated MOC fibers in chinchilla and obtained significant CAP reductions of up to 11 dB (for 2 kHz tones) accompanied by much smaller CM increases of <2.5 dB (Elgueda et al., 2011). The lesser efferent effects on CMs than on CAPs have also being reported for sustained contralateral noise that elicits sizable CAP suppressions and only small CM enhancements (Larsen and Liberman, 2009). In contrast, auditory cortex deactivation in chinchilla, in most cases, produced decreases in both CM and CAP responses (León et al., 2012). In that case, as mentioned by the authors, the effects may have involved not only the activity of medial, but also lateral OC efferent fibers.

### Comparison of suppression in anesthetized and awake animals

Contralateral-sound suppression of cochlear responses has been mostly studied in anesthetized animals and it is known that the activity of medial OC neurons is dependent on the level of anesthesia (Liberman and Brown, 1986). Accordingly, to assess the physiological importance of this suppression effect it is important to measure it in awake animals. A study that compared contralateral-sound suppression of DPOAEs in anesthetized and awake guinea pigs found that suppression was much weaker in urethane-anesthetized than in awake animals, and that it was even weaker in pentobarbitone-anesthetized animals (Guitton et al., 2004). In this study we have compared the contralateral suppression of CAP responses in anesthetized and awake chinchillas finding that the mean values of suppression were higher by 1–3 dB in the awake than in the anesthetized condition (see Figure 8). These results add to previous evidence (Boyev et al., 2002; Guitton et al., 2004; Chambers et al., 2012) showing that when comparing the strength of efferent suppression measured in awake human subjects with that in experimental animals one must keep in mind that the latter is mostly measured in anesthetized animals and, consequently, is consistently underestimated.

### Frequency tuning of contralateral suppression

In all chinchillas, in anesthetized and awake condition, we found that the contralateral-tone CAP suppression was frequency tuned; that is, suppression had a peak for contralateral tones at frequencies equal or near those of the ipsilateral tones and decreased at a fast-rate for higher and lower frequencies (Figures 5–7). This tuning of contralateral suppression indicates a correspondence between the tonotopic distribution of afferent and efferent cochlear neural fibers. Afferent fibers from a contralateral cochlear location with a certain characteristic frequency activate MOC efferent neurons that innervate ipsilateral cochlear locations having similar characteristic frequencies. The close correspondence that we have found between the most effective contralateral frequencies and ipsilateral frequencies (Figure 9) is similar to that reported for auditory-nerve responses in cat (Warren and Liberman, 1989b). However, in the cat the most effective contralateral frequencies displayed a slight deviation from ipsilateral frequencies for ipsilateral tones at frequencies less than 3 kHz while in our case the deviation was for ipsilateral frequencies ≥4 kHz. These results are also in agreement with anatomical data of single olivocochlear neurons in cat and guinea pig showing that the cochlear region innervated by an efferent neuron corresponds (or is close) to the place innervated by afferent neurons with the same characteristic frequency (Robertson and Gummer, 1985; Liberman and Brown, 1986). The widths of the frequency tuning curves for CAP suppression that we obtained in chinchilla would correspond to efferent innervation spans of about 4 to 8% of total cochlear length; these values are in between those obtained by fiber labeling in cat (2.2–11.2%) by Liberman and Brown (1986) and in guinea pig (<1–2% and 1.4–7.8%) by Robertson (1984) and Robertson and Gummer (1985), and Brown (1987), respectively.

Our finding of narrow frequency tuning curves for contralateral CAP suppression, although in good agreement with suppression tuning curves previously obtained for auditory-nerve and CAP responses in anesthetized cats (Liberman, 1989; Warren and Liberman, 1989b), are in contradiction with two completely different frequency-selectivity characteristics of suppression recently reported using OAEs in humans. Stimulus frequency OAE measurements in humans indicate that the strength of contralateral suppression depends on the integration of the effect from almost the entire length of the contralateral cochlea (Lilaonitkul and Guinan, 2009). On the other hand, spontaneous OAE measurements indicate that contralateral suppression is tuned to a fixed narrow frequency band, between 500 and 1,000 Hz, independent of the spontaneous OAE frequency (Zhao and Dhar, 2012). As mentioned above, the narrow tuning of MOC effects that we have observed agrees well with previous evidence on cochlear efferent innervation, while the results obtained using different types of OAEs to assess MOC-effect tuning in humans are contradictory and difficult to interpret.

### Controls to discard any middle-ear involvement in suppressive effects

As mentioned above, there is solid experimental evidence indicating that the suppressive effects of contralateral sounds on cochlear responses are mediated by efferent uncrossed MOC fibers (Warren and Liberman, 1989a). However, there is always a possibility, especially when using high levels of stimulation, that the suppressive effects on CAP responses could be partially produced by activation of the middle-ear reflex that also reduces cochlear sensitivity. Several facts assure us that the CAP-amplitude reductions observed in these experiments were due to activation of the MOC system and not the middle-ear reflex. First, all contralateral suppressions (other than those in CAP input-output curves) were measured at or below 70 dB SPL intensity levels, and the strongest suppression effects were always found at much lower levels. Second, no differences in contralateral CAP reduction were found in one control animal in which the CAP-suppression frequency tuning was measured before and after middle-ear muscle section (Figure 11). Third, in the three awake chinchillas in which we did find efferent effects on CMs, contralateral sounds produced CAP reductions concomitant with CM enhancements. The presence of these opposite effects of contralateral stimulation on CAP and CM potentials is strong evidence that they were not produced by middle-ear reflex activation (Guinan, 1996; Robles and Delano, 2008). Fourth, the fact that CAP suppressions produced by contralateral tones were always sharply tuned to frequencies equal or close to that of the ipsilateral tone discards any possibility that they could be a consequence of middle-ear reflex activation.

In conclusion, we have shown that: (1) In spite of the lower percentage of uncrossed MOC fibers present in chinchilla, contralateral sounds produce consistent CAP amplitude reductions for 1–6 kHz ipsilateral frequencies. (2) The contralateral tones producing the CAP suppression are frequency tuned to a frequency equal or close to that of the ipsilateral tone. (3) The contralateral produced CAP suppressions correspond to a span of uncrossed MOC fiber innervation reaching at least the central third of the chinchilla cochlea. (4) The contralateral CAP suppression is consistently higher in awake than in anesthetized condition.

## Conflict of interest statement

The authors declare that the research was conducted in the absence of any commercial or financial relationships that could be construed as a potential conflict of interest.
